# FACTORS ASSOCIATED WITH SEVERITY OF DEPRESSION: EVIDENCE FROM THE CHINA HEALTH AND RETIREMENT LONGITUDINAL STUDY

**DOI:** 10.1093/geroni/igz038.1890

**Published:** 2019-11-08

**Authors:** Mingyang Zheng, Xian Liu

**Affiliations:** 1 University of Minnesota - Twin Cities, Minneapolis, Minnesota, United States; 2 Adelphi University, Garden City, New York, United States

## Abstract

Depression among older adults in China is widespread. To explore factors associated with depression among older adults, most studies focus on the individual as the unit of analysis. However, since individuals are nested in families, it is important to understand depression within a family context. To address this gap, the current study examines the degree to which individual and dyad-level characteristics were associated with the severity of depression among older couples in China. Data for the study were drawn from wave 4 of the China Health and Retirement Longitudinal Study. The total sample size in the study was 2560 older couples aged 60 and above. Multilevel modeling was used to analyze the dyadic data. Our preliminary findings suggested the partial intraclass correlation between a dyad’s depression scores was 0.32, which shows that a couple’s scores were similar to one another. Those who were female, were younger, lived in rural areas, had lower cognition, and those whose spouses had lower cognitive ability were associated with more severe depression. The findings provided empirical evidence to support the argument that more community mental health resources should be allocated to rural areas in China. Moreover, given that female older adults are more vulnerable to depression compared to male older adults, it is imperative to develop tailored services to support women to enhance their psychological wellbeing. In addition, as spousal cognition was negatively correlated with depression, services to support older couples where one has dementia are needed.

